# Characteristics of people with epilepsy and Neurocysticercosis in three eastern African countries–A pooled analysis

**DOI:** 10.1371/journal.pntd.0010870

**Published:** 2022-11-07

**Authors:** Dominik Stelzle, Veronika Schmidt, Luise Keller, Bernard J. Ngowi, William Matuja, Gabrielle Escheu, Peter Hauke, Vivien Richter, Emilio Ovuga, Bettina Pfausler, Erich Schmutzhard, Action Amos, Wendy Harrison, Joyce Kaducu, Andrea S. Winkler

**Affiliations:** 1 Center for Global Health, Department of Neurology, School of Medicine, Technical University of Munich, Munich, Germany; 2 Centre for Global Health, Institute of Health and Society, University of Oslo, Oslo, Norway; 3 Department of Neurology, School of Medicine, Technical University of Munich, Munich, Germany; 4 National Institute for Medical Research, Muhimbili Medical Research Centre, Dar es Salaam, Tanzania; 5 University of Dar es Salaam, Mbeya College of Health and Allied Sciences, Mbeya, Tanzania; 6 Department of Neurology, Muhimbili University of Health and Allied Sciences, Dar es Salaam, Tanzania; 7 Department of Neurology, Kliniken Ostallgaeu-Kaufbeuren, Kaufbeuren, Germany; 8 Department of Radiology, University Hospital Tuebingen, Tuebingen, Germany; 9 Department of Mental Health, Gulu University, Gulu, Uganda; 10 Department of Neurology, Medical University of Innsbruck, Innsbruck, Austria; 11 National Epilepsy Association Malawi, International Bureau of Epilepsy, Lilongwe, Malawi; 12 Department of Infectious Disease Epidemiology, Imperial College London, London, United Kingdom; 13 Ministry of Health, Kampala, Republic of Uganda; International Atomic Energy Agency, AUSTRIA

## Abstract

**Background:**

Neurocysticercosis (NCC), a zoonotic disease caused by the pork tapeworm *T*. *solium*, represents one of the most common causes of secondary epilepsy but remains often undiagnosed due to lack of awareness and diagnostic facilities.

**Methodology:**

We pooled data from four cross-sectional studies on epilepsy and NCC in eastern Africa. Study sites were in Uganda, Malawi and in Tanzania (Dar es Salaam and Haydom). The study in Uganda and Malawi were community-based, the two studies in Tanzania were hospital-based. The same questionnaire was used for assessment of clinical characteristics of patients with epilepsy. Computed tomography (CT) scans and serological testing were performed in order to diagnose NCC.

**Results:**

Overall, 1,179 people with epilepsy were included in our analysis. Of those, 941 PWE underwent CT scanning and were pooled for NCC analysis. Seventy patients were diagnosed with NCC, but NCC prevalence differed considerably between sites ranging from 2.0% (95%CI 0.4% to 3.6%) in Dar es Salaam to 17.5% (95%CI 12.4% to 22.6%) in Haydom. NCC prevalence did not show any association with sex but increased with age and was higher in rural than urban settings. In addition, being a farmer, non-Muslim, eating pork and living with pigs close by was associated with a higher NCC prevalence. PWE with NCC experienced their first epileptic seizure around 3 years later in life compared to PWE without NCC and their epileptic seizures seemed to be better controlled (p<0.001). There was no difference between focal onset seizures and focal signs on neurological examination in both groups (p = 0.49 and p = 0.92, respectively). The rT24H-EITB had a sensitivity for the detection of NCC of 70% (95% confidence interval [CI] 51 to 84%), the LLGP of 76% (95%CI 58 to 89%) and the antigen ELISA of 36% (95% CI 20 to 55%).

**Conclusions:**

NCC is prevalent among PWE in eastern Africa, although it may not be as common as previously stated. Demographic characteristics of PWE with NCC differed from those without NCC, but semiological characteristics and results on neurological examination did not differ compared to PWE without NCC. Interestingly, seizures seemed to be less frequent in PWE with NCC. Being aware of those differences and similarities may help triaging PWE for neuroimaging in order to establish a diagnosis of NCC.

## Author summary

Neurocysticercosis is a neglected zoonotic disease caused by the pork tapeworm *Taenia solium*. Patients with neurocysticercosis show a wide range of symptoms, from asymptomatic to severely affected by epileptic seizures, progressive headache or focal neurological deficits. Epileptic seizures are particularly common and up to 30% of people with epilepsy have neurocysticercosis in areas endemic for *Taenia solium*. The disease is often undiagnosed in Africa because of a lack of diagnostic opportunities (serological tests and neuroimaging). Furthermore, symptoms are too unspecific to diagnose patients solely based on clinical characteristics. Moreover, radiological features of neurocysticercosis differ between continents and have not been described yet for African populations. In this study, for the first time, we describe radiological but also clinical features of patients with epilepsy and neurocysticercosis in eastern Africa. We found that in Tanzania, Malawi and Uganda, neurocysticercosis is common in people with epilepsy although less common than previously reported. Also, most patients with neurocysticercosis have multiple parenchymal lesions and clinical features of people with epilepsy and neurocysticercosis do not differ substantially from people with epilepsy without neurocysticercosis.

## Introduction

Epilepsy is among the most common neurological disorders with approximately 46 million people affected globally [1]. This number will increase steeply, if family members acting as caregivers to people with epilepsy (PWE) will be included, as their lives, well-being, and productivity are indirectly affected. The majority of epilepsy cases are seen in low-income and middle-income countries (LMIC) [2]. However, there seems to be a wide variation of epilepsy prevalence estimates which may be due to the varied causes of epilepsy including genetic factors but also environmental and behavioural risk factors, like head trauma, being exposed to tapeworm carriers and the lack of hygiene; the latter two representing classical risk factors for the acquirement of *Taenia solium* neurocysticercosis (NCC) [3–5]. In some parts of Africa, NCC is considered the most common cause of secondary epilepsy, yet data on NCC prevalence are still scarce and endemicity of *T*. *solium* is mostly only inferred from porcine cysticercosis studies [3]. NCC is a poverty-associated classical One Health disease and seldomly diagnosed because of lack of awareness among medical personnel, limited diagnostic facilities in endemic areas, costly diagnostic tests, and lack of One Health disease surveillance at the community level, where diseased animals could be used to track NCC-affected individuals. Furthermore, even where available, serological tests for the diagnosis of *T*. *solium* NCC only have limited accuracy, particularly for single lesions and lesions located in the extraparenchymal space for which the tests often are false negative [6]. For this reason, most people with NCC in sub-Saharan Africa have unfortunately been diagnosed only in the context of scientific research projects and not in the general health care system. However, individual research projects are often based on small source populations and use different study designs and diagnostic methodologies, hence the NCC prevalence estimates may vary. This can only be overcome by large enough source populations.

Therefore, this study pools the population numbers and obtained research data recruited from four large studies in eastern sub-Saharan Africa. It furthermore aims to analyse the NCC prevalence of PWE and compare the geographic, demographic, clinical (past and present), semiological (related to epileptic seizures), serological (related to NCC diagnosis), neuroradiological and therapeutic (related to anti-seizure medication) characteristics of PWE with and without NCC. Furthermore, we assessed the diagnostic accuracy of three in-house tests for the detection of *T*. *solium* cysticercosis-specific antigen and antibodies.

## Methodology

### Ethics statement

Ethical clearance was obtained for all studies in the respective study country and Germany (where appropriate). Tanzania (both for Dar es Salaam and Haydom): National Institute of Medical Research in Dar es Salaam (Ref. No.MU/DRP/AEC/VOL. XIII/64); Directorate of Research and Publications, Muhimbili University of Health and Allied Sciences (MUHAS), Dar es Salaam (Ref. No.: MU/DRP/REC/Vol.I/36, MU/RP/AEC/Vol.XII/86 and MU/DRP/AEC/Vol.XVI/91); the Ethics Committee of Ludwig-Maximilians University (LMU) Munich, Germany, stated that clearance in Germany from the LMU was not necessary (correspondence from 23082008). Malawi: Malawi National Health Science Research Committee, NHSRC Ref. No. 910; Imperial College Research Ethics Committee, Ref. No. ICREC_11_3_6; Ethics Committee of the Technical University of Munich Ref. No. 3088/10. Uganda: Uganda National Committee for Science and Technology, UNCST Ref. No. 543; the Ethics Committee of Ludwig-Maximilians University (LMU) Munich, Germany, stated that clearance in Germany from the LMU was not necessary (correspondence from 23082008). Informed oral consent was obtained from all participants included in each study. In case of minors, oral consent was obtained from a parent or legal guardian. No further ethical clearance was necessary for the secondary data analysis.

The reporting followed the STROBE checklist (S1 Text).

**Fig 1:**
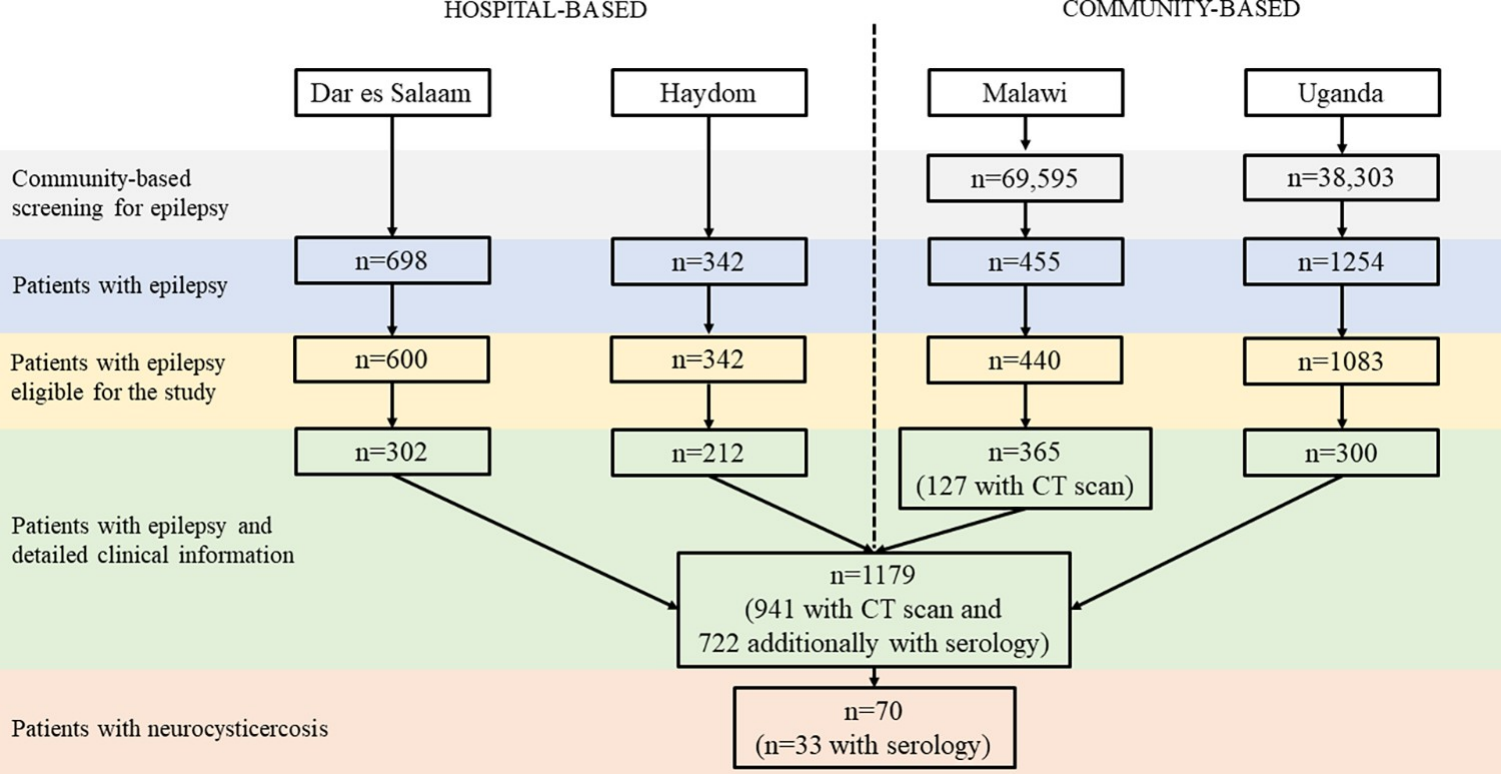
Flowchart of the patient selection by site^⸸^. ⸸ Detailed serological tests were available from the studies in Dar es Salaam, Malawi and Uganda.

For this analysis, we pooled demographic, clinical, semiological (related to epileptic seizures), serological (related to NCC diagnosis), neuroradiological, and therapeutic (related to anti-seizure medication) characteristics of PWE recruited from four different studies on epilepsy and NCC in eastern sub-Saharan Africa, including a community-based screening of 107,898 individuals in total [4]. Two studies were conducted in Tanzania, both in hospital-based settings [3,7]. One of these studies was conducted in Haydom in rural northern Tanzania and the other study in Dar es Salaam. The other two studies were community-based of which one was conducted in rural northern Uganda and the other one in rural southern Malawi [8]. Fig 1 shows the flowchart of PWE who were included in these four studies. In the following, the four studies are briefly summarised. In all studies, epilepsy was defined as two or more afebrile seizures unrelated to acute metabolic disorders or withdrawal of drugs or alcohol. Only individuals with epileptic seizures satisfying this definition and consenting to the different study procedures were included in the different studies. People with obvious reasons for their epilepsy such as perinatal hypoxic brain injury, traumatic brain injury, or a history of chronic alcohol misuse were excluded a priori from the studies. There was a cut-off for the recruitment of children in all studies which was based on a) the necessity to exclude children with febrile seizures and b) the opinion of the local ethics committee as to when it would be clinically justified for children to undergo radiation from computed tomography (CT) examination. The age cut-offs were in Dar es Salaam 5 years, in Haydom 11 years, in Malawi 6 years and in Uganda 12 years. All recruited PWE were examined neurologically and completed an in-depth questionnaire on present and past medical history with a focus on seizure history and semiology.

### Tanzania–Dar es Salaam

The study aimed to assess the prevalence of NCC in an urban population of PWE. Between March 2012 and December 2013, 698 consecutive PWE were identified from six governmental health centres and dispensaries in Kinondoni district, Dar es Salaam. All patients aged six years and older meeting the following criteria were eligible for the study: Onset of epilepsy above the age of five years to exclude febrile seizures, no relevant history of traumatic brain injury, and no past or present history of any substance abuse. Due to financial restrictions, not all remaining 600 PWE could be included. Hence, 302 of the 600 PWE were selected randomly for inclusion into the study and underwent further clinical evaluation, including neuroimaging. A detailed description of the recruitment process has been published elsewhere [7].

### Tanzania–Haydom

The study aimed to assess the prevalence of NCC in a rural population of PWE. Between August 2002 and November 2004, 342 consecutive patients were diagnosed with epilepsy by a neurologist (ASW) and recruited into the Haydom Lutheran Epilepsy Clinic, Mbulu district, Manyara region, for regular follow-up. In 2005, PWE from the Haydom Lutheran Epilepsy Clinic were contacted for inclusion in the study either during a follow-up appointment in the Epilepsy Clinic or by mail. All patients aged 11 years and older and meeting the above-mentioned inclusion/exclusion criteria were eligible for the study. Overall, 212 of the original 342 PWE could be recruited and underwent further clinical evaluation, including neuroimaging. Detailed information on the recruitment is published elsewhere [3].

### Malawi

This study aimed to assess the risk of precipitating neurological side effects associated with latent NCC following mass drug administration of praziquantel for the control of schistosomiasis. Between October 2012 and December 2013, the study was conducted in the rural Balaka district as a community-based, door-to-door screening study of nearly the entire population of the areas Chiyendausiku, Kalembo, and Mbera. Detailed study procedures have been published elsewhere.[8] The selection of the areas was based on the geographic presence of porcine cysticercosis as a sentinel for human disease. Before mass drug administration, nearly 70,000 people were screened for epileptic seizures using a 15-item questionnaire (S1 Table). In total, 3,100 people screened positive for epileptic seizures of which 1,913 were followed-up and 455 were diagnosed with epilepsy. All patients younger than 6 years of age, and those who only had epileptic seizures during childhood were excluded amounting to 440 people in total. Furthermore, 75 were lost to follow-up or were not willing to be included. The remaining 365 PWE underwent further clinical evaluation. Due to financial restrictions, not every patient could receive a CT scan. Patients were selected according to the recency of onset of epilepsy and the results of the serological test for *T*. *solium* cysticercosis (127 PWE). All patients with onset of epilepsy within the five years preceding the survey were offered a CT scan (n = 108) and among people with epilepsy onset longer than five years ago, everybody with a positive serology (n = 7) and a random subset of 14 people with negative serology.

### Uganda

The study aimed to assess the prevalence of NCC in a rural population of PWE. Between May 2010 and March 2011, a two-step random-cluster door-to-door, community-based study was conducted in three rural districts of northern Uganda (Adjumani, Gulu, and Moyo) based on the geographic presence of porcine cysticercosis as a sentinel for human disease. Almost 40,000 people were screened for epileptic seizures using a 9-item questionnaire (S1 Table). In total, 1,254 individuals who screened positive and were 12 years and above were eligible for the next phase of the study. After in-depth examination and application of the inclusion and exclusion criteria as described above, 1,083 were eligible for the study. Due to financial restrictions, 300 patients were selected according to the recency of onset of epilepsy and underwent further clinical evaluation, including neuroimaging.

### Evaluation of epilepsy and neurocysticercosis

In all studies, the same in-depth epilepsy questionnaire was used, with only few questions that were not included in all four studies (indicated as not applicable [NA] in the tables). Data were collected on demography, seizure history and semiology, comorbidities, past medical and psychiatric history, perinatal and family history, anti-seizure medication, and neurological examination.

Furthermore, patients underwent serological testing for *T*. *solium* specific antibodies in all four study sites and *T*. *solium* specific antigens in three study sites. In the studies in Dar es Salaam, Malawi and Uganda lentil lectin purified glycoprotein enzyme-linked immunoelectrotransfer blot (LLGP-EITB) and rT24H-immunoblot [9,10] were performed for *T*. *solium* cyst specific antibodies and B158/B60-ELISA [11] for *T*. *solium* cyst specific antigen. In the study in Haydom, a commercially available cysticercosis western blot (LDBiodiagnostics, Lyon, France) for the detection of antibodies was used.

LLGP-EITB is an enzyme-linked immunoelectrotransfer blot that detects cysticercosis-specific antibodies for seven glycoproteins [10,12]. One of the seven diagnostic bands—the glycoprotein (Gp) 50 band—potentially cross-reacts with other parasitic infections such as *T*. *saginata*, *Echinococcus granulosus*, and *Schistosoma spp*. [10,13]. The rT24H-test is an immunoblot that detects cysticercosis-specific antibodies to a *T*. *solium* recombinant antigen.[9, 10] Cross-reactions are described with *Entamoeba histolytica*, *Hymenolepis nana* and *Schistosoma spp*..[9] The ELISA for *T*. *solium* antigen in serum is a monoclonal antibody (B158/B60 antibodies) capture-based ELISA and was performed following a modified protocol as described in Dorny et al. [11]. This test may cross-react with other *Taenia spp*., such as *T*. *saginata*. Serological tests were performed at the Division of Parasitic Diseases and Malaria, Center for Global Health, Centers for Disease Control and Prevention (CDC), Atlanta, Georgia, USA. Patients were categorised as serologically positive for *T*. *solium* if any of the aforementioned tests was positive. Additionally, the rES33-EITB was performed in combination with the rT24H-EITB for the detection of *T*. *solium* taeniosis-specific antibodies in Dar es Salaam, Malawi and Uganda [10]. The rES33-test is an immunoblot that detects adult *T*. *solium* specific-antibodies using a recombinant protein derived from the excretory-secretory proteins of the adult tapeworms [14]. Furthermore, patients received a CT scan. CT scanning was undertaken locally at the various study sites and supervised by trained clinical personnel. CT scans were all read by the same neuroradiologist (VR) at the Department of Neuroradiology, Technical University of Munich (TUM), Germany.

Seizure type was classified according to the 2017 ILAE definition of seizure onset (focal/generalised) [15]. We based the diagnosis solely on semiological characteristics; if patients reported an aura before seizures or a unilateral seizure onset, the seizure was classified as focal onset otherwise as generalised onset. We also classified the patients according to the epilepsy definition by Winkler et al. [16] which was created for African settings. For this classification, all available information (apart from CT scan) was taken into consideration. Diagnosis of NCC was based on the 2017 updated Del Brutto criteria [17]. NCC was classified as definite or probable according to neuroimaging, and clinical/exposure criteria. All patients included in this analysis were considered to have both minor clinical/exposure criteria: Clinical manifestations suggestive of NCC and coming from or living in an area where cysticercosis is endemic. The stage of the NCC lesions was categorized as active (vesicular/colloidal/granular nodular stage), inactive (calcified stage), or mixed (both active and inactive lesions present). The location of the cysts was classified as parenchymal, extraparenchymal or both mixed (parenchymal/extraparenchymal).

### Statistical analyses

Baseline data were presented by centre and as total values. Numbers and proportions were presented for binary variables; continuous data were presented as the median and interquartile range (IQR). Although screening and examination tools were well comparable between studies, we mostly refrained from statistical testing. Where reasonable, Chi-square tests were run for between centre differences, and for patients with and without NCC. All analyses were performed using R version 4.1.1.

## Results

**Fig 2:**
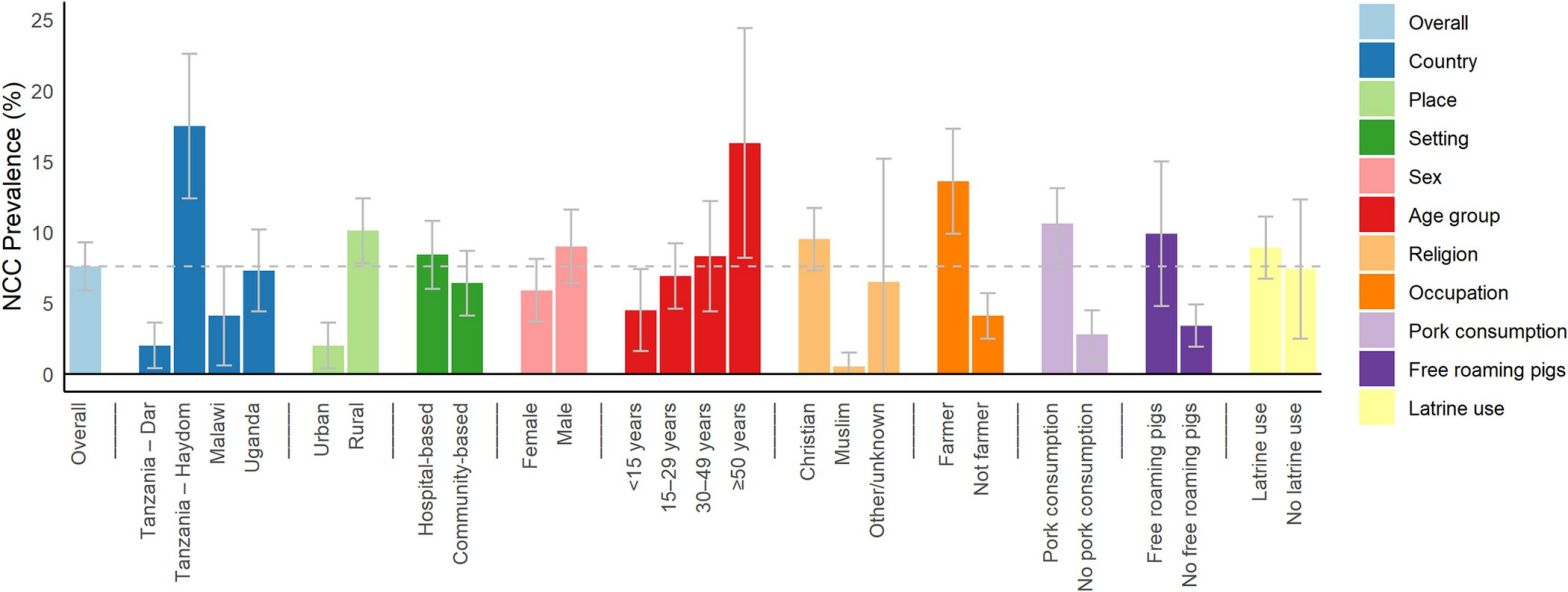
Neurocysticercosis prevalence disaggregated by study-specific and socio-demographic factors.

Overall, 2,749 PWE were included in the four studies of which 1,179 had an in-depth clinical assessment and 941 received a CT scan (and 722 additionally had serological tests performed). Among the 941, 70 (7.6%) patients were diagnosed with NCC in the four studies together; 36 (51%) of them had a definite diagnosis and 34 (49%) a probable diagnosis according to the updated 2017 Del Brutto criteria (Fig 1 and Table 1). 17 of the 34 patients with probable diagnosis did not have serological test results. NCC prevalence differed by study site and ranged from 2.0% in Dar es Salaam (urban; hospital-based) to 17.5% in Haydom (rural; hospital-based; p<0.001 for the difference between studies). NCC prevalence was higher in the rural study sites (10.0%) compared with the urban study site (2.0%; p<0.001); in the hospital-based settings, NCC prevalence was 8.4% and 6.3% in the community-based settings (p = 0.23). NCC was more common among males than females (8.9% versus 5.9%), albeit this association was not statistically significant (p = 0.10). NCC prevalence increased with age (p = 0.009) from 4.5% among children (<15 years) to 16.3% among people aged 50 years and older. Muslim people were less commonly affected by NCC compared with people of other religions (p<0.001). Patients who eat pork, and patients who have free-roaming pigs close by more commonly had NCC (p<0.001, p = 0.003). The use of a latrine was not associated with NCC prevalence (p = 0.76, Fig 2). Two patients (3%) with NCC had only active lesions, 18 (26%) had mixed (active and inactive) lesions, and 50 (71%) had only inactive lesions (Table 2). The majority of people with NCC had more than three lesions (n = 49, 70%), and NCC lesions were mostly located in the parenchyma (n = 61, 87%). All extraparenchymal lesions were located in the subarachnoid space and none in the ventricular system. However, intraventricular cysts can hardly be detected with only CT imaging; signs/symptoms that may indicate intraventricular cysts are hydrocephalus and severe headache. In our epilepsy population, only few patients had hydrocephalus, and none was associated with *T*. *solium* positive serology, which makes it rather unlikely that many intraventricular cysts were not detected. Children mostly had calcified lesions (2/12) and 7 out of 14 children with NCC had at least three lesions.

**Table 1:** Baseline characteristics of people with epilepsy from four different studies.

		Tanzania (Dar es Salaam)	Tanzania (Haydom)	Malawi	Uganda	Overall	Overall (with CT available)
		Urban	Rural	Rural	Rural		
		Hospital-based	Hospital-based	Community-based	Community-based		
n		302	212	365	300	1179	941
Sex	female	160 (53.0)	104 (49.1)	189 (51.8)	128 (42.7)	581 (49.3)	460 (48.9)
	male	142 (47.0)	108 (50.9)	176 (48.2)	172 (57.3)	598 (50.7)	481 (51.1)
Age group	children (<15 years)	58 (19.2)	42 (19.8)	103 (29.3)	57 (19.0)	260 (22.3)	203 (21.6)
	adolescents (15–29 years)	148 (49.0)	113 (53.3)	141 (40.2)	154 (51.3)	556 (47.7)	462 (49.1)
	adults (30–49 years)	73 (24.2)	38 (17.9)	77 (21.9)	61 (20.3)	249 (20.6)	194 (20.6)
	elderly (≥50 years)	23 (7.6)	19 (9.0)	30 (8.5)	28 (9.3)	100 (8.6)	82 (8.7)
Age in years	median (IQR)	23 [16–32]	22 [15–30]	21 [13–32]	21 [15–32]	22 [15–32]	22 [15–32]
Marital status	single	232 (76.8)	126 (59.4)	232 (63.6)	58 (19.3)	648 (54.9)	226 (24)
	married/cohabiting	54 (17.9)	81 (38.2)	92 (25.2)	55 (18.3)	282 (23.9)	51 (5.4)
	seperated/divorced/widowed	14 (4.6)	5 (2.4)	38 (10.4)	24 (8)	82 (6.9)	497 (52.8)
	unknown	2 (0.7)	0 (0)	3 (0.8)	163 (54.3)	168 (14.2)	167 (17.7)
Religion	Christian	151 (50.0)	189 (89.2)	215 (58.9)	284 (94.7)	839 (71.2)	707 (75.1)
	Muslim	148 (49.0)	3 (1.4)	147 (40.3)	6 (2.0)	304 (25.8)	199 (21.1)
	other	0 (0.0)	6 (2.8)	3 (0.8)	10 (3.3)	19 (1.6)	18 (1.9)
	unknown	3 (1.0)	14 (6.6)	0 (0.0)	0 (0.0)	17 (1.4)	17 (1.8)
Occupation	farmer	0 (0)	196 (92.5)	179 (49.0)	91 (30.3)	466 (39.5)	334 (35.5)
	professional	59 (19.5)	0 (0)	7 (1.9)	3 (1)	69 (5.9)	66 (7.0)
	student	93 (30.8)	0 (0)	103 (28.2)	0 (0)	196 (16.6)	149 (15.8)
	trader/business	3 (1)	5 (2.4)	15 (4.1)	9 (3)	32 (2.7)	20 (2.1)
	other	15 (5)	8 (3.8)	27 (7.4)	197 (65.7)	247 (20.9)	226 (24.0)
	none	123 (40.7)	3 (1.4)	14 (3.8)	0 (0)	140 (11.9)	134 (14.2)
	unknown	9 (3)	0 (0)	20 (5.5)	0 (0)	29 (2.5)	12 (1.3)
Pork consumption		155 (51.7)	162 (77.5)	103 (29)	213 (71.2)	637 (54.0)	568 (61.1)
Family pork consumption		184 (61.3)	87 (41.0)	NA	NA	271 (52.9)	271 (52.9)
Free roaming pigs living close by		23 (7.7)	NA	58 (16)	86 (28.8)	167 (14.2)	134 (18.6)
Latrine use		221 (90.6)	203 (97.1)	NA	220 (73.6)	644 (85.6)	644 (85.6)

**Table 2:** Computed tomography findings in people with epilepsy and neurocysticercosis.

		Radiological findings–n (%)			
		All	Children (<15 years)	Adults (≥15 years)	Time since onset of epileptic seizures in years (mean [95% CI])
Total		70	14	56	
Stage	active lesions	2 (3)	1 (7)	1 (2)	4 [2.5–5.5]
	mixed (active/inactive) lesions	18 (26)	1 (7)	17 (30)	10.8 [3.3–9.8]
	inactive lesions	50 (71)	12 (86)	38 (68)	7.7 [3–9.8]
Number of lesions	1	14 (20)	2 (14)	11 (20)	
	2 or 3	7 (10)	5 (36)	2 (4)	
	> 3	49 (70)	7 (50)	42 (75)	
Location	parenchymal	61 (87)	12 (86)	49 (88)	
	extraparenchymal	4 (6)	2 (14)	2 (4)	
	mixed (parenchymal/extraparenchymal)	5 (7)	0 (0)	5 (9)	

CI confidence interval

Among PWE with NCC, epilepsy started in the median 3 years later in life and proportionally more people were older than 21 years when epileptic seizures started compared to PWE without NCC (Table 3). Also, people with already calcified lesions, had epileptic seizures for a longer period of time (Table 2). In addition, PWE with NCC had epileptic seizures less frequently and epilepsy was less active (both p<0.001), even though there was no difference in the proportion of PWE who were on anti-seizure medication (p = 0.23). Focal onset of seizures and focal neurological signs/symptoms on neurological examination were not associated with NCC (p = 0.49 and p = 0.92, respectively).

**Table 3:** Clinical characteristics of people with epilepsy with and without neurocysticercosis.

		PWE with NCC n (%)	PWE without NCC n (%)
n		70	871
Age seizures started	median (interquartile range), years	17 (11–35)	14 (11–23)
	≤21 years	41 (58.6)	637 (73.4)
	>21 years	29 (41.4)	231 (26.6)
Frequency of seizures	daily to weekly	1 (1.6)	52 (6.7)
	weekly to monthly	1 (1.6)	68 (8.8)
	monthly to yearly	21 (33.9)	410 (53.0)
	yearly or less often	39 (62.9)	243 (31.4)
Time since last seizure	<1month	11 (16.9)	230 (31.3)
	1–3 months	5 (7.7)	123 (16.7)
	3–12 months	10 (15.4)	88 (12.0)
	1–2 years	12 (18.5)	87 (11.8)
	>2 years	27 (41.5)	208 (28.3)
Most occurence of seizures	anytime	18 (28.1)	426 (51.4)
	daytime	16 (25.0)	179 (21.6)
	evening/night	12 (18.8)	95 (11.5)
	night while asleep	18 (28.1)	129 (15.6)
Loss of consciousness		65 (92.9)	848 (97.9)
Motor activity; side of body	bilateral	54 (79.4)	698 (87.4)
	unilateral	13 (19.1)	91 (11.3)
	none	1 (1.5)	10 (1.3)
Anti-seizure medication		62 (88.6)	711 (82.1)
Phenobarbital		21 (34)	245 (34)
Phenytoin		14 (23)	138 (19)
Carbamazepine		27 (44)	295 (41)
Valproic acid		0 (0)	4 (1)
Combination therapy		0 (0)	29 (4)
Focal neurological signs on examination		1/6 (16.7)	22/308 (7.1)
Diagnosis ILAE	generalised onset	54 (77.1)	717 (82.2)
	focal onset	16 (22.9)	152 (17.5)
	unclassified	0 (0.0)	3 (0.3)
Diagnosis according to Winkler et al.[16]	generalised seizures within a specific age range	34 (47.2)	434 (49.9)
	generalised seizures outside specific age range	22 (30.6)	177 (20.4)
	generalised seizures with diffuse brain damage	2 (2.8)	115 (13.2)
	generalised seizures with focal signs	12 (16.7)	130 (15.0)
	simple partial seizures	2 (2.8)	6 (0.7)
	complex partial seizures	0 (0.0)	4 (0.5)
	unclassified	0 (0.0)	3 (0.3)

PWE People with epilepsy, NCC Neurocysticercosis, ILAE International League Against Epilepsy

### Serological results and neurocysticercosis

**Fig 3:**
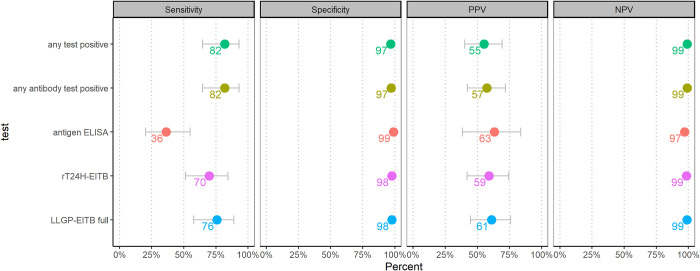
Test performance and findings on computed tomography.

Detailed serological results were available of patients recruited through the studies in Dar es Salaam, Malawi, and Uganda. Overall, 722 patients from these studies had detailed serological and CT scan results and among those, 33 patients were diagnosed with NCC. At least one serological test (LLGP-EITB, rT24H-EITB or Antigen ELISA) was positive for 27/33 (82%) NCC patients. In 6/33 (18%) people with NCC, all tests were negative. Serological tests were more often positive for patients with active or mixed stage NCC lesions compared with only calcified lesions (12/13 [94%] versus 15/20 [75%]). Patients with more than 3 lesions also were more commonly positive in any serological test (23/25 [92%] versus 4/8 [50%]). Whilst 22/26 (85%) and 5/5 (100%) patients with parenchymal or mixed (parenchymal and extraparenchymal) lesions were positive in any serological test, both patients with exclusively extraparenchymal lesions were negative in all serological tests (0/2; 0%; Fig 3). LLGP-EITB had a higher sensitivity (25/33, 76%) than the rT24H-EITB (23/33, 70%). The tests performed the same for active or mixed stage lesions, but for calcifications LLGP-EITB was better. Antigen ELISA was positive in 12/33 (36%) patients with any type of NCC, but for 9/13 (69%) patients with active or mixed stage NCC lesions. Test accuracy differed for children (≤18 years) and adults. The sensitivity was higher for all tests for adults compared with children: LLGP-EITB was 88% (22/25) for adults and 38% (3/8) for children, the rT24H-EITB was 80% (20/25) compared with 38% (3/8) and the sensitivity of antigen ELISA was 48% (12/25) for adults and 0% (0/8) for children. At the same time, the specificity was higher for all tests for children (S3 Table). 11 of the 33 (33%) people with NCC were positive in the rES33-EITB, 9/13 (69%) patients with active or mixed stage NCC lesions and 2/20 (10%) patients with calcified lesions (Table 4). No child was positive in the rES33 but 11/25 (44%) adults with NCC (S3 Table).

**Table 4:** Serological results by stage, number, and location of neurocysticercosis-typical lesions.

		NCC	Stage of lesions		Number of lesions		Location of lesions				
		Overall	Active or mixed	Inactive	≤3 lesions	>3 lesions	Parenchymal	Extraparen-chymal	Mixed parenchymal and extraparenchymal	No NCC	Total
Overall		33	13	20	8	25	26	2	5	689	722
*T*. *solium* cysticercosis antibodies or antigen	All tests negative	6 (18)	1 (8)	5 (25)	4 (50)	2 (8)	4 (15)	2 (100)	0 (0)	667 (97)	673 (93)
	Any test positive	27 (82)	12 (92)	15 (75)	4 (50)	23 (92)	22 (85)	0 (0)	5 (100)	22 (3)	49 (7)
*T*. *solium* cysticercosis antibodies	negative	6 (18)	1 (8)	5 (25)	4 (50)	2 (8)	4 (15)	2 (100)	(0)	669 (97)	675 (93)
	positive	27 (82)	12 (92)	15 (75)	4 (50)	23 (92)	22 (85)	0 (0)	5 (100)	20 (3)	47 (7)
*LLGP-EITB*	negative	8 (24)	1 (8)	7 (35)	6 (75)	2 (8)	6 (23)	2 (100)	0 (0)	673 (98)	681 (94)
	positive	25 (76)	12 (92)	13 (65)	2 (25)	23 (92)	20 (77)	0 (0)	5 (100)	16 (2)	41 (6)
rT24H-EITB*	negative	10 (30)	1 (8)	9 (45)	7 (87)	3 (12)	8 (31)	2 (100)	0 (0)	673 (98)	683 (95)
	positive	23 (70)	12 (92)	11 (55)	1 (13)	22 (88)	18 (69)	0 (0)	5 (100)	16 (2)	39 (5)
*T*. *solium* cysticercosis antigen ELISA	negative	21 (64)	4 (31)	17 (85)	7 (87)	14 (56)	18 (69)	2 (100)	1 (20)	682 (99)	703 (97)
	positive	12 (36)	9 (69)	3 (15)	1 (13)	11 (44)	8 (31)	0 (0)	4 (80)	7 (1)	19 (3)
*T*. *solium* taeniosis antibodies: rES33-EITB*	negative	22 (67)	4 (31)	18 (90)	8 (100)	14 (56)	19 (73)	2 (100)	1 (20)	680 (99)	702 (97)
	positive	11 (33)	9 (69)	2 (10)	0 (0)	11 (44)	7 (27)	0 (0)	4 (80)	9 (1)	20 (3)

* the rT24H and rES33 were analysed in a single combined EITB; LLGP lentil lectin purified glycoprotein; EITB enzyme-linked immunoelectrotransfer blot

## Discussion

In this study, we pooled the results of four studies on epilepsy and NCC in eastern Africa to describe the clinical characteristics of PWE and the burden of NCC among them. We furthermore analysed the accuracy of 3 serological in-house tests for the diagnosis if *T*. *solium* NCC.

### Clinical characteristics of people with epilepsy with neurocysticercosis

We were able to demonstrate that NCC was common in PWE, especially in rural areas with the highest value of 17.5% in Haydom, Tanzania. However, NCC prevalence was lower than previously reported. A meta-analysis from 2020 found a prevalence of NCC among PWE of 22% (95%CI 17% to 27%) in sub-Saharan Africa with large variations between regions and countries [18]. However, variation between villages were also reported. A study in Burkina Faso found an NCC prevalence among PWE ranging from 0% in a mainly Muslim village with nearly no pigs, to close to 50% in two pig rearing villages [19]. It is this large variation within small areas that makes it difficult to report overall estimates of NCC prevalence as the burden may differ from one village to another. NCC is a poverty-related One Health zoonotic disease. It is more common in areas where hygiene is poor and where people live closely with free-roaming pigs, which is also demonstrated by our results [20–22]. NCC was more frequent among farmers, non-Muslims, people eating pork and was positively associated with age; findings that are in line with previously published literature [23–25]. However, NCC was prevalent also among young people (<15 years; almost 13% in our NCC population) which means infection may occur at a very young age already. The observed correlation of NCC prevalence with age does not allow for inferences about trends in infections, as the end stage of NCC is often calcifications that remain lifelong in the brain. Even an assessment of infection trends evaluating only patients with active lesions is challenging as often there are years between infection and symptom onset and some patients may never become symptomatic at all [26]. This makes it difficult to trace back the route of infection. However, the low proportion of patients with active stage lesions indicates that infection occurred long time ago which may point to an improvement of the prevention of *T*. *solium* infection. The relatively low prevalence of NCC among PWE in Malawi and Uganda suggest that *T*. *solium* may not be highly endemic in the studied districts. This could be because pig keeping is not as common as in the other sites, because pigs roam freely less often or because education programmes had been in place which prevented the transmission of *T*. *solium*. The pig keeping business with freely roaming pigs was particularly flourishing around Haydom for some years prior and during the study, which may also explain the high NCC prevalence in PWE there. In all study areas, NCC was a relatively unknown disease to the local populations and many clinicians before they got in contact with our study.

In our study, most patients had parenchymal and multiple lesions which is partly different from India and Latin America, where the predominant features are single parenchymal lesions of the granuloma type and a mixture of multiple parenchymal but also extraparenchymal lesions, respectively [26,27]. This may be due to complex interactions between the *Taenia solium* parasite, its host(s) and environmental factors such as infection pressure, which has previously been reported [28–30]. It also needs to be mentioned that the radiological features as described in our study may be biased by the lack of highly sensitive CT scanners, those available in sub-Saharan Africa often are of mediocre quality, and limited access to MRI scanners. Therefore, extraparenchymal presentations may have gone unnoticed.

Studies showed that clinical characteristics of PWE with NCC are similar to those without NCC except for NCC patients more commonly having focal onset seizures and developing seizures later in life [7,31]. In our pooled analysis, PWE with NCC had their first seizure later in life, and proportions of focal onset of seizures or presence of focal neurological signs on examination was higher among PWE with NCC, although this association was statistically not significant (Table 3). Interestingly and importantly, seizures frequency seemed to be better controlled in PWE with NCC compared to those without NCC. This does not seem to be due to anti-seizure medication as the proportion of PWE on treatment was comparable in both groups (between 80% and 90%; Table 3). Nonetheless, seizure frequency was lower among PWE with NCC. Clinical characteristics, in general, were not so substantially different although demographic variables may give some clue (i.e., PWE from rural settings, non-Muslim farmers who eat pork and live nearby pigs, and people with seizure onset in adulthood). These factors do not allow for a diagnostic algorithm to select people for neuroimaging in areas where neuroimaging is not available everywhere and not affordable for all. However, such an algorithm would be needed to reduce the costs for the diagnosis of NCC.

Symptomatic NCC in active stages, be it intra- or extraparenchymal (in the subarachnoid space), should be treated with anthelmintic medication (single or in combination) together with preceding as well as concomitant corticosteroids. Treatment is rarely an emergency as NCC often remains silent for several years before it causes symptoms. Nonetheless, treatment is important because studies showed that through anthelmintic medication cysts may resolve entirely and not remain as calcifications [32,33]. The reason why people with NCC develop epileptic seizures is hypothesized to be due to inflammation that develops around lesions when the immune system reacts to the cysticercus [34–36]. However, epileptic seizures may still occur in patients with only inactive lesions remaining. This may be due to calcifications being an epileptogenic focus themselves, or due to the continued expression of *T*. *solium* antigen which may cause oedema around calcifications periodically [37–39]. Either way, this has implications for anti-seizure medication. In our study, half of the people with NCC had only inactive lesions and nonetheless still epileptic seizures despite being treated with anti-seizure medication. This may be due to under-dosing, non-compliance, or choice of inappropriate anti-seizure medication. In terms of anti-seizure medication discontinuation, it has been recommended that seizure-free patients after resolution of all active lesions should remain on treatment for at least two years before anti-seizure medication can be levelled off [40].

### Serology in people with epilepsy with neurocysticercosis

In our study, nearly every fifth PWE with NCC was serologically negative for *T*. *solium* antigen and antibody. Many of these patients had only calcified lesions which means they may have been serologically positive at some point but became negative after a while. At the same time, some patients were serologically positive, but did not have any NCC-typical lesion visible on CT scan. This may either be because not every patient with cysticercosis has NCC, that NCC lesions were not detected on CT due to poor quality or that serological tests were false positive, possibly due to cross-reactions described earlier with *T*. *saginata*, *Echinococcus granulosus*, *Echinococcus granulosus* or *Schistosoma spp*. [9,13]. The sensitivity and specificity of the LLGP-EITB was similar as previously reported in a laboratory study that analysed serum of people with NCC from Peru [10]. In that study, LLGP-EITB had a sensitivity of 96% to detect ≥2 viable cysts and 52% for one viable cyst. In our study, the small number of patients with active or mixed stage NCC did not allow for this differentiation by number, but 12 out of 13 (92%) patients with active or mixed stage NCC were positive in the LLGP-EITB. The specificity in our study was 97%, as in the study with the Peruvian serum samples [10].

Furthermore, we found that the in-house LLGP-EITB had the highest sensitivity of all tests used in the 3 studies for the accuracy analysis. Whilst the rT24H performed equally well as the LLGP-EITB for active lesions (both 92% sensitivity), for inactive lesions the test performed worse (55% sensitivity compared with 75%). In the study by Noh et al. the rT24H performed also only slightly worse [10].

In general, in our study, serological tests performed better for adults than for children. This may be influenced by the location, stage, and number of lesions which were distributed differently between adults and children. Among children, ten of 12 patients had only inactive lesions which highlight an infection at a very young age. In general, patients with only calcified lesions less often were serologically positive in our population.

The low sensitivity of the antigen ELISA comes from the large number of patients with only calcified lesions. This has previously been described. The presence of antigen decreases as the cysts develop from active-stage lesions into calcifications. However, this means also that antigen ELISA is a good biomarker for detecting active-stage lesions, a stage during which the patient may benefit from anthelmintic therapy. Studies have also shown that antigen ELISA can be used to assess treatment success in patients with active lesions [41].

Despite inaccuracies of the tests, serological testing may be useful in supporting NCC diagnosis, although it is rarely available due to the lack of inexpensive and easy-to-perform commercial tests and trained laboratory personnel. Point-of-care tests, which are currently under development, could improve the situation in time.

### Strengths and limitations

This study had several strengths but also limitations. The collection of data on PWE with NCC as presented in this manuscript is exceptional, spanning work of over 10 years and pooling results of four studies in three countries of East Africa. We describe the different characteristics of almost 1,200 PWE and the burden of NCC among those with CT examination (almost 950 PWE), which is unique within the sub-Saharan African setting. Hence, this work adds substantially to the body of literature on epilepsy and NCC globally, and specifically to the evidence on NCC burden in PWE and the associated demographic, clinical, semiological, serological, neuroradiological, and therapeutic characteristics of PWE with and without NCC in sub-Saharan Africa. This has policy implications in the respective countries, i.e. Tanzania, Uganda, and Malawi, and further supports data on the ever-growing burden of neurological disorders in LMIC [42,43]. In addition, results from the present analysis contribute valuable information to the WHO Intersectoral Global Action Plan on Epilepsy and other Neurological Disorders which is currently being drafted [44] and to the Roadmap for Neglected Tropical Diseases 2030 [45] and therefore also contributes to policy at a global level.

However, our analyses also have limitations. One limitation is that although the studies used the same methods for the epilepsy assessment and neurological examination, the examinations were conducted by different people and the questionnaire was translated to different local languages. This may have affected the results. However, demographic factors or questions regarding seizure onset, frequency, or seizures–variables that we focussed on in our analyses–were probably less affected than questions with more subjective answers. Whilst different CT scanners were used in the different countries, the scans were evaluated by the same radiologist (VR), which makes the results reliable and comparable. However, neuroimaging was only performed with CT and not as recommended with a combination of CT and magnetic resonance imaging (MRI). MRI detects active lesions better than CT, whereas calcifications are better seen on CT compared to MRI [46]. Hence, the prevalence of active-stage NCC lesions may have been underestimated in our study.

Another limitation was the design of the four studies, two were hospital-based and two were community-based. This means that the populations are not necessarily comparable since not all patients suffering from epileptic seizures are attending mental health clinics due to various reasons such as (fear of) stigmatization or distance to the next health care facility. The difference is probably that more symptomatic patients seek treatment and may therefore be overrepresented in the hospital-based studies. However, also in the community-based studies, patients received further neurological follow-up including CT, if they screened positive in a screening questionnaire for epileptic seizures. Stigma and only slight signs/symptoms, e.g. very few epileptic seizures, may also have affected the recruitment process [20]. Where possible in our analysis, we have pointed out the differences between hospital-based and community-based studies. In general, there were not many differences apart from anti-seizure medication, and we therefore consider the data appropriate for pooled analyses.

## Conclusion

In this study, we pooled demographic, clinical, semiological, serological, neuroradiological, and therapeutic findings of four studies (serological results only from three studies) in PWE with and without NCC. NCC is common in PWE in rural pig-keeping areas where *T*. *solium* is endemic and hygiene is poor, but probably not as common as previously stated, ranging from 2% in urban to almost 20% in rural areas. Although epilepsy in PWE with NCC starts later in life compared to those without NCC, children and adolescents are affected too (around 13% of our NCC population). Interestingly, PWE with NCC seem to respond better to anti-seizure medication than PWE without NCC although treatment varied between countries. There was a trend towards focality in PWE with NCC (focal onset seizures and focal signs on neurological examination), but this was not significant. NCC represents a curable cause of epilepsy, yet diagnosis remains challenging as it requires neuroimaging which is expensive and not affordable for most people who are affected. To date, neither clinical algorithms nor point-of-care tests for *T*. *solium* are available to streamline selection for neuroimaging. Thus, nearly all people with NCC are being diagnosed within research projects and a large proportion of symptomatic people with NCC likely remains undiagnosed and therefore untreated. Our study results further underline the need for appropriate policy around PWE with NCC at the local and global level, in addition to context-specific management guidelines with a focus on resource-limited settings.

## Supporting information

S1 Text STROBE Statement—Checklist of items that should be included in reports of cross-sectional studies. (DOCX)

S1 Table Epilepsy screening questionnaire Malawi. (DOCX)

S2 Table Epilepsy screening questionnaire Uganda. (DOCX)

S3 Table Serological test results and neurocysticercosis lesions, for adults and children. (DOCX)
