# Correction: Characteristics of people with epilepsy and Neurocysticercosis in three eastern African countries–A pooled analysis

**DOI:** 10.1371/journal.pntd.0011101

**Published:** 2023-01-26

**Authors:** Dominik Stelzle, Veronika Schmidt, Luise Keller, Bernard J. Ngowi, William Matuja, Gabrielle Escheu, Peter Hauke, Vivien Richter, Emilio Ovuga, Bettina Pfausler, Erich Schmutzhard, Action Amos, Wendy Harrison, Joyce Kaducu, Andrea S. Winkler

The Funding statement is incomplete. It should read as follows: This study was funded by the German Research Foundation (DFG) [https://www.dfg.de/] within the research grant (WI 3427/1-1) “Neurocysticercosis in sub-Saharan Africa” (ASW) and by the Bill and Melinda Gates Foundation [https://www.gatesfoundation.org/] (ASW, WH) within the research grant (1017886) “Integrated Control of Cysticercosis in sub-Saharan Africa”. Dominik Stelzle was supported by the Federal Ministry of Education and Research, Germany, Grant 01KA2112B, within the Cysticercosis Network of Sub-Saharan Africa (CYSTINET-Africa). The funders had no role in the detailed study design, data collection and analysis, decision to publish, or preparation of the manuscript.
