# Supplementary material for: Characteristics of people with epilepsy and Neurocysticercosis in three eastern African countries–A pooled analysis
Source: PLoS Negl Trop Dis. 2022 Nov 7;16(11):e0010870. doi: 10.1371/journal.pntd.0010870 (PMC9639810; doi:10.1371/journal.pntd.0010870)
Supplement: S1 Table — (DOCX) [file pntd.0010870.s002.docx]

S1 Table. Epilepsy screening questionnaire Malawi

| 1. | Have you ever lost consciousness or fallen due to lost consciousness? |
| --- | --- |
| 2. | Have you ever been told that while you were unconscious your arms and legs shake or stretch out? |
| 3. | Have you had attacks in which you fall and bite your tongue or lost control of your bladder or bowels? |
| 4. | Have you had uncontrollable attacks of shaking or trembling in one arm or leg or in the face without losing consciousness? |
| 5. | Have you had attacks in which you lose contact with the surroundings without losing consciousness? |
| 6. | Have you ever been told that you had episodes of strange behaviour without remembering it? |
| 7. | Have you ever been told that you had epilepsy or epileptic seizures? |
| 8. | Did you/your child have seizures between one month and 7 years of age? |
| 9. | Was there a fever with all of the attacks? |
| 10. | Did the seizures continue after the age of 7? |
| 11. | How old were you when you had your first attack (enter YY:MM)? |
| 12. | Have you had an attack in the last 4 weeks? |
| 13. | How long ago in months was your last attack? |
| 14. | How often do you have these attacks?  1 Daily 2 Once a week or more but less than daily 3 Once a month or more but less than once a week 4 Once every six months or more but less than once a month  5 Once a year or more but less than once every six months  6 Less than once a year 7 Not applicable |
| 15. | Do you take antiepileptic medication? |
