# Supplementary material for: Characteristics of people with epilepsy and Neurocysticercosis in three eastern African countries–A pooled analysis
Source: PLoS Negl Trop Dis. 2022 Nov 7;16(11):e0010870. doi: 10.1371/journal.pntd.0010870 (PMC9639810; doi:10.1371/journal.pntd.0010870)
Supplement: S2 Table — (DOCX) [file pntd.0010870.s003.docx]

S2 Table. Epilepsy screening questionnaire Uganda

| 1. | Have you ever lost consciousness or fallen due to lost consciousness? |
| --- | --- |
| 2. | Have you ever been told that while you were unconscious your arms and legs shake or stretch out? |
|  | Have you ever had attacks in which you fell and bit your tongue or lost control of your bladder or bowels? |
|  | Have you ever had uncontrollable attacks of shaking or trembling in one arm or one leg, or in the face without losing consciousness? |
|  | Have you ever experienced attacks of numbness, tingling in one arm or one leg without losing consciousness? |
|  | Have you ever had attacks in which you lose contact with the surroundings without losing consciousness? |
|  | Have you ever had attacks of losing awareness that was associated with a feeling of vagueness, unreality or dreaminess or experience of abnormal smells, sounds, or vision without losing or before loss of consciousness? |
|  | Have you ever been told that you had episodes of strange behaviour without remembering it? |
|  | Have you ever been told that you have or had epilepsy or epileptic seizures? |
