# Supplementary material for: Characteristics of people with epilepsy and Neurocysticercosis in three eastern African countries–A pooled analysis
Source: PLoS Negl Trop Dis. 2022 Nov 7;16(11):e0010870. doi: 10.1371/journal.pntd.0010870 (PMC9639810; doi:10.1371/journal.pntd.0010870)
Supplement: S3 Table — (DOCX) [file pntd.0010870.s004.docx]

S3 Table. Serological test results and neurocysticercosis lesions, for adults and children

|  |  | Adults | | Children | |  |
| --- | --- | --- | --- | --- | --- | --- |
|  |  | NCC | No NCC | NCC | No NCC | Total |
| Overall |  | 25 | 410 | 8 | 279 | 722 |
| *T. solium* cysticercosis antibodies or antigen | All tests negative | 1 (4) | 390 (95) | 5 (63) | 277 (99) | 673 (93) |
|  | Any test positive | 24 (96) | 20 (5) | 3 (38) | 2 (1) | 49 (7) |
| *T. solium* cysticercosis antibodies | negative | 1 (4) | 392 (96) | 5 (63) | 277 (99) | 675 (93) |
|  | positive | 24 (96) | 18 (4) | 3 (38) | 2 (1) | 47 (7) |
| *LLGP-EITB* | negative | 3 (12) | 396 (97) | 5 (63) | 277 (99) | 681 (94) |
|  | positive | 22 (88) | 14 (3) | 3 (38) | 2 (1) | 41 (6) |
| *rT24H-EITB** | negative | 5 (20) | 396 (97) | 5 (63) | 277 (99) | 683 (95) |
|  | positive | 20 (80) | 14 (3) | 3 (38) | 2 (1) | 39 (5) |
| *T. solium* cysticercosis antigen ELISA | negative | 13 (52) | 404 (99) | 8 (100) | 278 (100) | 703 (97) |
| *T. solium* cysticercosis antibodies or antigen | positive | 12 (48) | 6 (1) | (0) | 1 (0) | 19 (3) |
| *T. solium* taeniosis antibodies:  rES33-EITB | negative | 14 (56) | 403 (98) | 8 (100) | 277 (99) | 702 (97) |
|  | positive | 11 (44) | 7 (2) | (0) | 2 (1) | 20 (3) |

* the rT24H and rES33 were analysed in a single combined EITB;

LLGP lentil lectin purified glycoprotein; EITB enzyme-linked immunoelectrotransfer blot
